# The significance of reactive oxygen species in the formation of calcium oxalate stones and the protective effects of antioxidants on the kidneys

**DOI:** 10.3389/fimmu.2025.1540075

**Published:** 2025-05-21

**Authors:** XiaoLong Ying, Yang Chen, ZongYao Hao, HaoRan Liu

**Affiliations:** ^1^ Department of Urology, The First Affiliated Hospital of Anhui Medical University, Anhui Medical University, Hefei, Anhui, China; ^2^ Institute of Urology, Anhui Medical University, Hefei, Anhui, China; ^3^ Anhui Province Key Laboratory of Urological and Andrological Diseases Research and Medical Transformation, Anhui Medical University, Hefei, Anhui, China

**Keywords:** kidney stones, reactive oxygen species, antioxidants, oxidative stress, renal

## Abstract

Exposure of renal tubular epithelial cells (RTCs) to kidney stones or calcium oxide crystals triggers the production of reactive oxygen species (ROS), leading to oxidative stress. This oxidative milieu incites cellular injury and elicits an inflammatory cascade within the RTCs. Notably, the cellular membranes of the compromised cells facilitate the adherence and subsequent retention of crystals, which is instrumental in the pathogenesis of kidney stones. The pathways of ROS production are diverse, involving numerous signaling cascades. Recent researchers’ endeavors have elucidated that selective antioxidants can attenuate intracellular ROS concentrations by modulating these intricate signaling cascades. This reduction in ROS levels has been empirically demonstrated to significantly curtail the accumulation of calcium oxalate crystals within renal tissues in animal models, heralding a novel therapeutic paradigm for the amelioration of nephrolithiasis. In this review, we endeavor to elucidate the contributory role of ROS in kidney stone and explore the protective mechanisms by which certain antioxidants safeguard renal function.

## 1 Introduction

Urolithiasis, a prevalent disorder within the urinary system, emerges as the third most common urological affliction, trailing only behind urinary tract infections and prostate-related disorders. This pathological state of biomineralization, primarily manifesting within the renal system, is characterized by the crystallization and solidification of urinary constituents. The primary manifestation of this condition, kidney stones, impinges upon approximately 10-15% of the global populace (1). Existing literature delineates a differential propensity for nephrolithiasis influenced by factors such as gender, ethnicity, and geographic localization. Contemporary studies underscore a notable ascendancy in prevalence amongst females, a trend potentially ascribed to shifts in dietary and lifestyle paradigms (2). Specifically, in Eastern nations such as China and Japan, the embracement of Western dietary practices, marked by elevated protein consumption, correlates with an amplified incidence of nephrolithiasis (3). Calcium oxalate (CaOx) stones, constituting the quintessential component of urinary calculi, account for upwards of 80% of all diagnosed cases (4). It is generally believed that the formation process mainly includes urine supersaturation, heterogeneous nucleation, crystal growth and aggregation, and crystal adhesion and deposition in renal tubular cell, though the precise mechanisms remain enigmatic. Despite the advent of minimally invasive surgical interventions, the recurrence of urinary stones persists at a formidable rate, with a 50% recurrence within a five-year span and a potential surge to 75% over a decade, thereby levying substantial burdens upon individuals and healthcare infrastructures alike (5). This backdrop accentuates the imperative for the delineation and deployment of efficacious therapeutic strategies for nephrolithiasis, with an emphasis on minimizing adverse effects.

Recent research has elucidated a significant correlation between the injury of RTCs, the insurgence of calcium oxalate (CaOx) crystals presence, and the concomitant inflammatory responses and stone formation, thereby illuminating their contributory role in the pathophysiology of nephrolithiasis. A pivotal factor in this process is the induction of oxidative stress (OS) through ROS, which significantly contributes to stone formation. The injury of RTCs membrane integrity engenders a conducive environment for calcium crystal adhesion, thereby precipitating further stone development. Oxidative stress typically results from an imbalance between ROS production and antioxidant defenses. Under physiological conditions, the levels of ROS production are low and can be cleared by the endogenous antioxidant system. In contrast, an overproduction of ROS, outstripping the scavenging capacity of antioxidants, leads to oxidative stress, contributing to stone development (6–8).

The origins of ROS within the human body are multifarious, entailing the involvement of an array of signaling pathways. Recent investigations suggest that modulating some of these pathways, such as the MEK-ERK pathway and redox-sensitive pathways implicated in viral infections, can reduce ROS levels *in vivo*, correlating with diminished stone formation in animal models (9). This review aims to dissect the role of ROS in the etiology of nephrolithiasis and assess the potential of antioxidants as therapeutic agents in the management of calcium oxalate kidney stones, underpinning the prospective therapeutic ramifications of oxidative stress mitigation in the prevention of stone recurrence.

## 2 ROS

### 2.1 Definition and production pathway of reactive oxygen species

ROS, including free radicals, atoms, or molecules with unpaired electrons and their metabolites, comprise oxygen radicals like superoxide anion radicals (O_2_•−) and hydroxyl radicals (•OH), as well as non-radical oxidants such as hydrogen peroxide (H_2_O_2_) and singlet oxygen (¹O_2_). ROS’s reactive nature facilitates its role in various regulatory processes and signal transduction pathways. These include regulating proliferation, activating or deactivating biomolecules, and controlling transcriptional activity. However, excessive ROS may cause harmful effects through oxidative modifications of cellular components, particularly damaging proteins, lipids, carbohydrates, and nucleotides (10).

**Figure 1:**
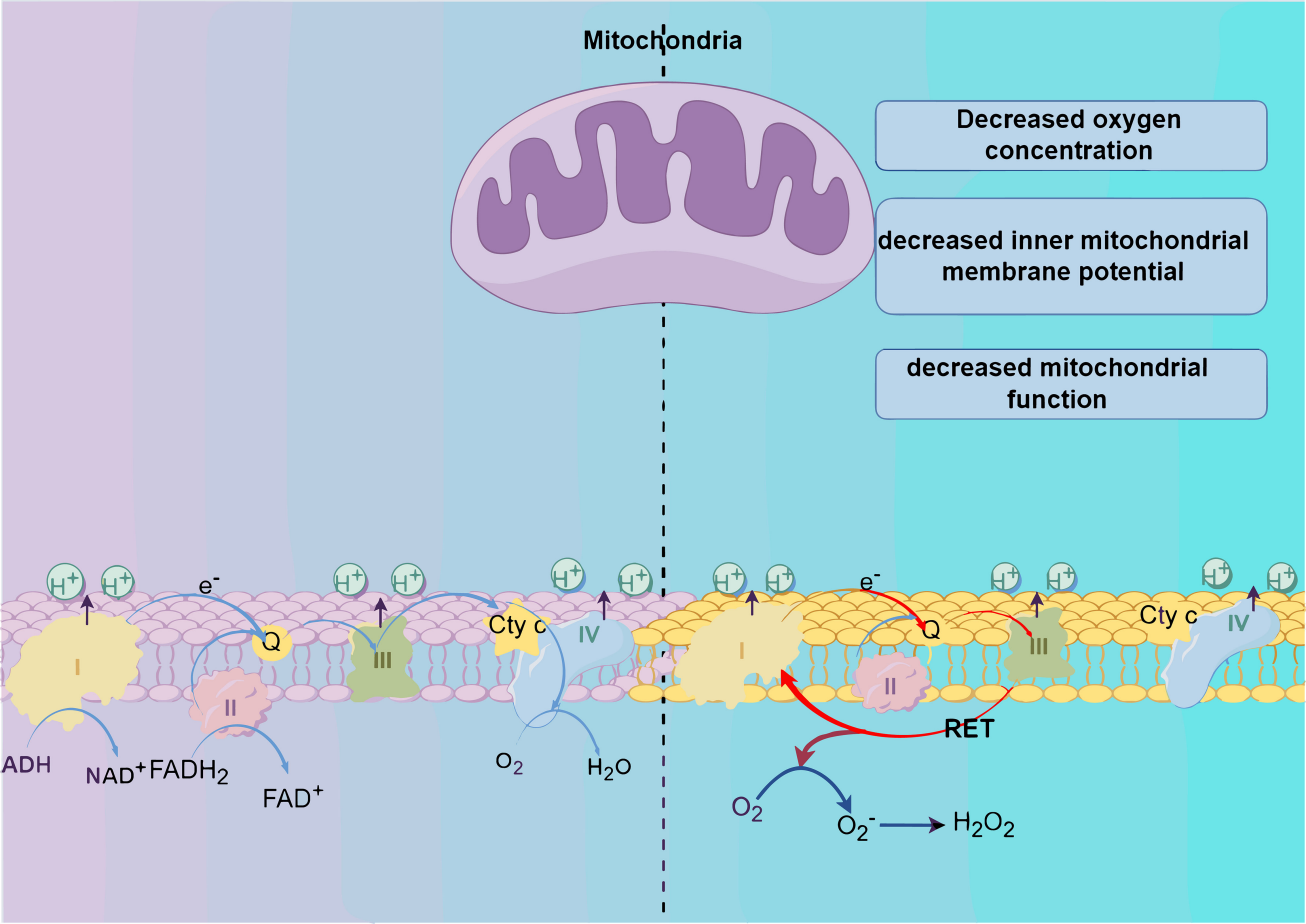
Production of reactive oxygen species (ROS). The generation of reactive oxygen species (ROS) primarily originates from the oxidative respiratory chains within mitochondria. Alterations in the internal environment, such as a decrease in the level of oxygen, mitochondrial membrane potential, or mitochondrial function, trigger reverse electron transfer (RET), facilitating electron circulation between C I and C II. Ultimately, this process culminates in the reaction of electrons with oxygen and subsequent ROS production.

The primary cellular sources of ROS include mitochondria and NADPH oxidase (NOX) (7). The electron transport chain (ETC), situated on the inner mitochondrial membrane (IMM), plays a crucial role within mitochondria. NADH and FADH2 introduce electrons into the ETC through complex I (CI) and complex II (CII). Electrons then move from complex I (CI) and complex II (CII) to complex III (CIII). Normally, electrons pass from CIII to complex IV (CIV) and combine with oxygen to form water. However, under specific conditions such as reduced oxygen levels, diminished mitochondrial membrane potential(MMP), or impaired mitochondrial function, electron transfer can become abnormal. Abnormal electron transport may result in the return of electrons from CIII to CI, which is known as reverse electron transfer (RET). Reverse electron transfer results in a cyclic flow of electrons moving back and forth between CI and CIII. During this cycle, interaction of the electrons with oxygen generates superoxide anion (O_2_•−). Subsequently, superoxide can undergo conversion into other forms of ROS, like hydrogen peroxide (H_2_O_2_) (11) (**Figure 1**).

In the cytoplasm, NADPH oxidases (NOXs) are crucial in maintaining cellular redox balance (12). The process begins with glucose metabolism through glycolysis, yielding pyruvate as the end product. This pyruvate is then transported to the mitochondria to enter the tricarboxylic acid (TCA) cycle. This process generates reducing equivalents, specifically NADH and FADH2, which subsequently enter the mitochondrial electron transport chain for oxidative phosphorylation. NOXs operate by facilitating the electron transfer from NADPH to oxygen, resulting in the generation of superoxide. This regulation of NOXs activity is controlled by transforming growth factor β (TGF-β) (13). Notably, *in vivo* exposure of renal tubular cells to oxalic acid triggers reactive oxygen species (ROS) production via the TGF-β1-NADPH-ROS pathway (14). Moreover, ROS generated by NOX can prompt mitochondrial ROS production, serving as a crucial pathway for ROS amplification or propagation (15). Given the high energy demands of the kidney, with its mitochondrial content and oxygen consumption being second only to the heart, RTCs are particularly vulnerable to damage from ROS imbalance, eventually leading to crystal accumulation and aggregation.

### 2.2 Physiological and pathological effects of reactive oxygen species

The role of ROS as crucial signaling molecules in cell proliferation and survival has been well established (16). At physiological levels, ROS facilitate cell proliferation and differentiation, making the maintenance of basal ROS levels essential for normal cell growth and development (17). Moreover, ROS act as signaling molecules that activate transcription factors and upregulate the expression of protective genes such as antioxidant enzymes, thus bolstering cellular resistance to oxidative stress (18). Additionally, ROS also interact with antioxidant molecules such as glutathione and peroxidase, playing a vital role in regulating intracellular redox homeostasis and thereby influencing cellular function. However, deviations from optimal ROS levels, either excessively high or low, can lead to detrimental effects.

Organisms have developed numerous antioxidant defense systems to mitigate damage from ROS. These defenses include non-enzymatic systems, such as vitamins C and E, carotenoids, reduced glutathione, and polyphenols, and enzymatic systems like superoxide dismutase (SOD), catalase (CAT), and glutathione peroxidase (GPX). Together, these systems synergistically safeguard cells against oxidative damage (19).

## 3 The role of ROS in the formation of calcium oxalate renal stones

It is now well-recognized that the injury to RTCs and subsequent formation of stones attributed to oxalate is due to the interaction of oxalate with the kidney generating ROS (20). Oxidative stress (OS) is defined as a state of excessive oxidation within an organism, primarily caused by either an overproduction or inadequate clearance of ROS in response to harmful stimuli in both the internal and external surroundings. This results in the accumulation of ROS within the body or cells, leading to oxidative damage and disruption of the body’s oxidative/antioxidant balance system. It manifests as harm to cellular membranes, proteins, DNA, and lipids (21).

Studies conducted *in vitro* on cells, *in vivo* on animals, and clinical case studies have all shown that RTCs exposed to oxalate and/or calcium oxalate crystals generate excessive ROS, triggering oxidative stress and inflammatory reactions. Prolonged oxidative stress exacerbates damage to these cells. As oxidative stress continues to exacerbate damage to RTCs, the ability for phosphatidylserine (PS), situated on the cell’s inner side, to move outward increases and its ability to move inward decreases, resulting in PS flipping to the cell surface. Phosphatidylserine (PS), as a phospholipid of the renal tubular epithelial cell membrane that can adsorb crystals, flips to the cell surface, enhancing the adhesiveness of stone crystals at the flipped sites (22).

**Figure 2:**
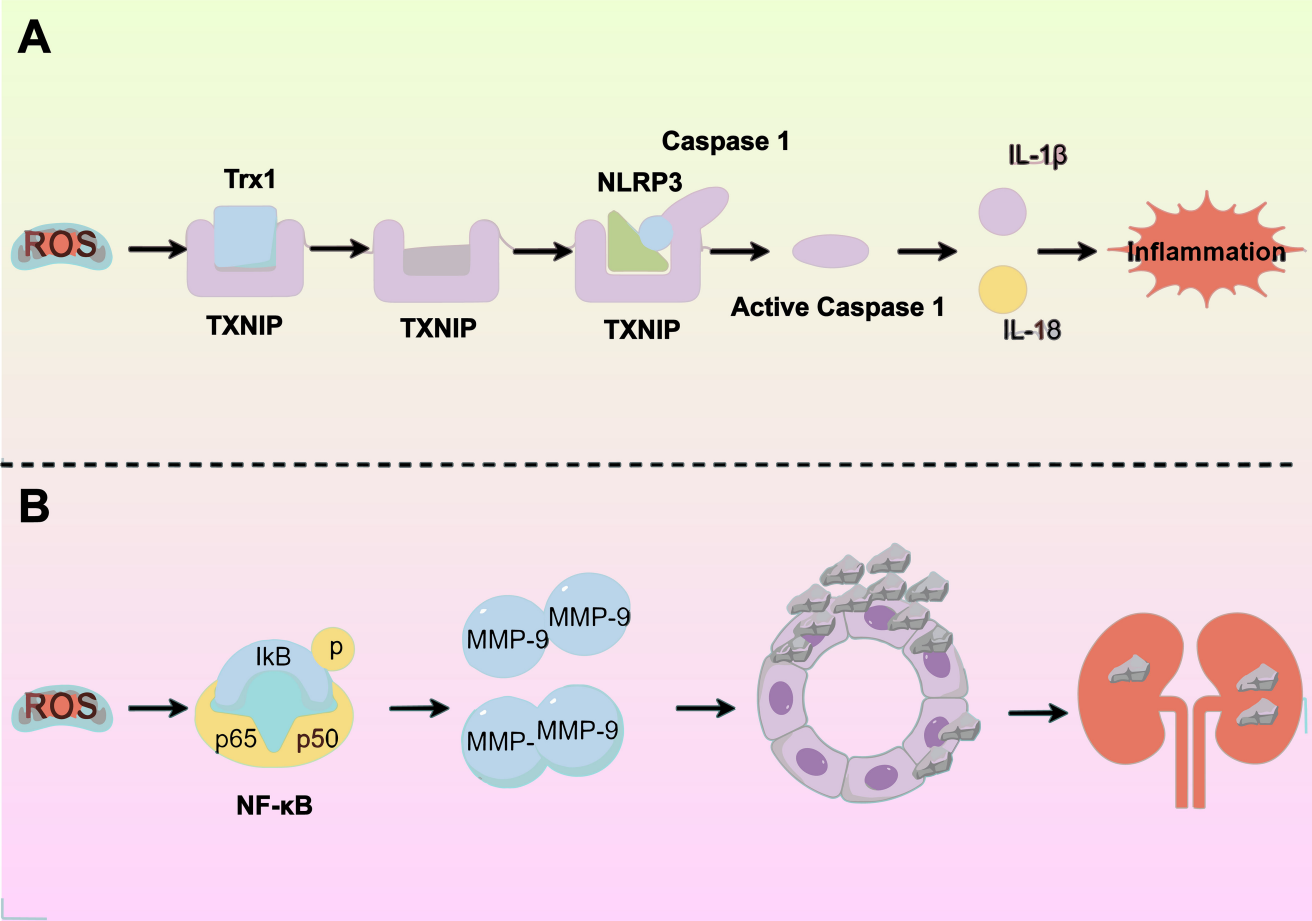
Exposure to oxalate and/or calcium oxalate crystals triggers renal tubular epithelial cells to produce excess reactive oxygen species (ROS), leading to oxidative stress (OS) and inflammatory responses. This process is driven by several molecular mechanisms: **(A)** Activation of the NLRP3 inflammasome releases inflammatory factors, worsening tubular epithelial damage. **(B)** Elevated ROS levels upregulate MMP-9 expression, increasing crystal adherence.

Joshi et al. discovered that a diet high in oxalate causes increased OS in the kidneys. They noted that under conditions of elevated ROS levels, intracellular TXNIP (thioredoxin-interacting protein) detaches from the antioxidant enzyme Trx1 (thioredoxin 1). This detachment leads to the activation of NLRP3, mediated by TXNIP (23). Activating the NLRP3 inflammasome releases inflammatory factors such as IL-1β and IL-18, triggering an inflammatory response. This response causes damage and necrosis in RTECs, setting the stage for CaOx stone formation (24). And increased ROS production can lower MMP, potentially causing mitochondrial and lysosomal dysfunction. This dysfunction can impair macrophages’ phagocytosis, reducing their capacity to eliminate calcium oxalate crystals during stone formation. Consequently, this can further encourage stone formation (25) (**Figure 2A**).

Additionally, Wiger et al. discovered that high ROS levels increase AP-1 (activator protein-1) and NF-κB (nuclear factor κB) levels, subsequently increasing the expression of matrix metalloproteinases (MMPs). MMPs, crucial components of the extracellular matrix (ECM), serve as effective crystal-binding molecules that play a significant role in the adhesion, internalization, and eventual formation of renal stones. Notably, MMP-9 facilitates dendritic cell migration, triggers the cleavage of the E-calmodulin receptor CD103 on dendritic cells, and encourages epithelial-mesenchymal transition, leading to sediment adhesion and aggregation. Additionally, MMP-9’s activity in degrading collagen and osteoblast cleavage products enhances inflammatory cell recruitment, a process linked to kidney stone development (3) (**Figure 2B**).

Furthermore, Under physiological conditions, urinary proteins including osteopontin and uromodulin (Tamm-Horsfall protein) inhibit kidney stone formation and support renal function (26, 27). However, elevated reactive oxygen species (ROS) under oxidative stress induce oxidative modifications of these protective proteins. Such redox alterations enhance calcium oxalate crystallization processes by promoting crystal nucleation, growth, and aggregation, thereby increasing lithogenic risk (28).

Tight junctions serve as crucial barriers in renal tubular epithelial cells, preventing the entry of water, ions, macromolecules, and pathogens into the renal tubules through the spaces between cells. Impairment of these tight junctions can obstruct intercellular channels, leading to damage of tubular epithelial cells and inciting inflammatory responses. This damage facilitates the accumulation of kidney stones within the tubules. Additionally, excessive production of ROS can degrade and redistribute tight junction proteins, such as ZO-1 and occludin, via the Akt/ASK1/p38 MAPK signaling pathway. This disruption of tight junction integrity contributes to stone formation (29).

Furthermore, the membranes of RTCs predominantly consist of phospholipids with unsaturated fatty acids. ROS have a high affinity for the unsaturated bonds in these fatty acids, leading to lipid peroxidation. This oxidative damage impairs the RTCs and enhance the adhesiveness of calcium oxalate stones to these cells. Consequently, this enhances the aggregation, nucleation, and growth of stone crystals in the kidneys.

## 4 Antioxidant

### 4.1 Sources and classification of antioxidants

Antioxidants are compounds that inhibit the oxidation of other compounds. The process of oxidation involves the transfer of electrons from a substance to an oxidizing agent, resulting in the generation of free radicals that initiate a cascade reaction. When this chain reaction occurs within a cell, it results in cellular damage or apoptosis. Antioxidants’ role is to eliminate free radicals, terminate chain reactions, and inhibit further oxidative reactions, while they themselves are oxidized (30, 31).

Antioxidants are abundant in daily life, with major sources including endogenously synthesized antioxidants, such as water-soluble and lipid-soluble antioxidants and antioxidative enzymes;natural antioxidants from food, such as various types of flavonoids, flavanols, anthocyanins, etc.; and common synthetic antioxidants such as butyl hydroxybenzoate (BHA), butylated hydroxytoluene (BHT), EDTA, etc. (32). Antioxidants are categorized into enzymatic antioxidants (including superoxide dismutase (SOD), catalase (CAT), glutathione peroxidase (GPx), and thioredoxin (Trx) systems) and non-enzymatic antioxidants (such as flavanes, phenols, and carotenoids) (33). Additionally, certain minerals like selenium and zinc function as antioxidants. These minerals are crucial components of antioxidant enzymes and are essential for maintaining their activity.

### 4.2 Mechanisms of antioxidant action

Antioxidants play a crucial role in the body by inhibiting oxidative reactions and reducing cellular damage. They can protect cell membranes; for example, vitamin E acts as a peroxide scavenger, capturing peroxyl free radicals to halt free radical chain reactions, which helps prevent lipid peroxidation (34). Rachel et al. demonstrated that antioxidants like superoxide dismutase (SOD) can convert superoxide into oxygen and hydrogen peroxide, thereby protecting cells against harm caused by free radicals (35).

Moreover, antioxidants affect various biological processes, including apoptosis, inflammation, and immune responses. For instance, vitamin C can inhibit endothelial cell oxidative stress induced by tumor necrosis factor-alpha (TNF-α), thus protecting vascular endothelial cells from damage (36). Additionally, phenolic compounds, another type of antioxidant, can halt the propagation of free radicals by exchanging protons with them (37). Furthermore, antioxidants can modulate the activity of inflammation-related transcription factors like NF-κB, thereby diminishing inflammatory responses (38). They also regulate the activity of other transcription factors such as AP-1, Sp-1, affecting gene expression (39).

While antioxidants offer numerous benefits to the body, it’s important to be mindful of their dual effects. At low concentrations, antioxidants protect against oxidative cell damage; however, at high concentrations, they may become toxic and disrupt cellular redox balance. Additionally, antioxidants can chelating metal ions, reducing their oxidizing capacity. Yet, this interaction might also result in increased levels of reactive hydroperoxides from these ions, eventually generating harmful free radicals (40). Consequently, the exploration and application of antioxidants in therapeutic contexts present significant challenges that need to be addressed.

## 5 The role of antioxidants in the treatment of kidney stones

### 5.1 The role of SOD in the treatment of kidney stones

Superoxide Dismutase (SOD) is a crucial antioxidant enzyme in cells, playing a pivotal role in shielding them from oxidative stress damage (41). There are three types of SOD: Cu/Zn-SOD (SOD1) found primarily in the cytoplasm, Mn-SOD (SOD2) located in mitochondria, and EC-SOD (SOD3) present in the extracellular matrix of mammalian tissues (42, 43). SOD1 catalyzes the reaction between superoxide (O_2_•−) and hydroperoxide (H_2_O_2_), aiding in the reduction of intracellular superoxide levels (44). SOD2 helps maintain intracellular redox balance by countering oxidative stress in mitochondria (45). They differ in their genetic structure, evolution, and expression patterns. These enzymes play a crucial role in organisms by helping to maintain oxidative balance and protect cells from the effects of oxidative stress.

Kang et al. discovered that increasing SOD activity significantly lowers the incidence of kidney stone formation and kidney injury, highlighting SOD’s ability to inhibit autophagy and endoplasmic stress response (46).The process of kidney stone formation is highly intricate, such as mitochondrial dysfunction, which disrupts energy metabolism, intracellular calcium ion balance, and the antioxidative defense system, and can lead to tubular cell damage, apoptosis, and the formation of stones through the combination of cellular inclusions and mitochondrial debris with calcium oxalate crystals in the tubular lumen (47). Antioxidant supplementation, by restoring the enzymatic activities of SOD, catalase (CAT), glutathione peroxidase, and glutathione (GSH), can prevent or mitigate the severity of stone deposits (48). Therefore, SOD, particularly Mn-SOD in mitochondria, plays a critical protective role against kidney stone formation.

### 5.2 The role of vitamin E in the treatment of kidney stones

Vitamin E, a fat-soluble antioxidant, plays a critical role in protecting polyunsaturated fatty acids within cell membranes from oxidative damage, thereby preserving cell membrane integrity (49). Known for its potent antioxidative properties, vitamin E counters free radical damage and has shown potential benefits in a range of diseases, including cancer, cataracts, Alzheimer’s disease, asthma, allergies, and diabetes. Specifically, Thamilselvan et al. demonstrated that vitamin E therapy can prevent calcium oxalate crystal deposition in the kidneys induced by hyperoxaluria, enhancing the antioxidant status of renal tissues (50). By trapping free radicals, vitamin E reduces their cytotoxic effects, which in turn helps protect cells from oxidative harm and may inhibit the attachment of calcium oxide apatite crystals, thereby preventing kidney stone formation (51). Furthermore, vitamin E is suggested to have a protective role in progressive renal failure, especially as oxidative stress exacerbates the progression of chronic inflammatory nephropathy (52).

### 5.3 The role of tea polyphenols in the treatment of kidney stones

Tea contains a collection of polycyclic phenolic compounds known as tea polyphenols, primarily consist of flavanols like catechins, along with flavan derivatives such as theaflavins (TF) and thearubigins (TR) (53). These compounds function as antioxidants by donating hydrogen atoms to inhibit free radical production, thus protecting cells from oxidative damage (54).

Research has found that NF-κB can regulate the expression of genes encoding adhesion molecules, COX-2, and inflammatory cytokines (such as TNF-α, IL-6, and CRP). These factors contribute to kidney stone formation by activating NADPH oxidase, which stimulates ROS production and impairs endothelial function through a vicious cycle mechanism [2]. Tea polyphenols counteract this by enhancing cellular antioxidant defenses, stimulating the Nrf2-Keap1-ARE signaling pathway, while suppressing the NF-κB signaling pathway (55). Catechins, the main substances in tea polyphenols, trap free radicals, thereby preventing oxidative stress-induced cellular damage. Their protective effect extends to inhibiting apoptosis caused by oxidative stress and chelating metal ions like copper and iron to reduce free radical production (56). Furthermore, Ye et al. found that theaflavins can up-regulate the expression of SIRT1, attenuating oxidative stress injury in the kidney induced by calcium oxalate. This phenomenon is partly attributed to the suppression of miR-128-3p, which normally suppresses SIRT1, suggesting new potential therapeutic targets for calcium oxalate kidney stones (57).

### 5.4 The role of sulforaphane in the treatment of kidney stones

**Figure 3:**
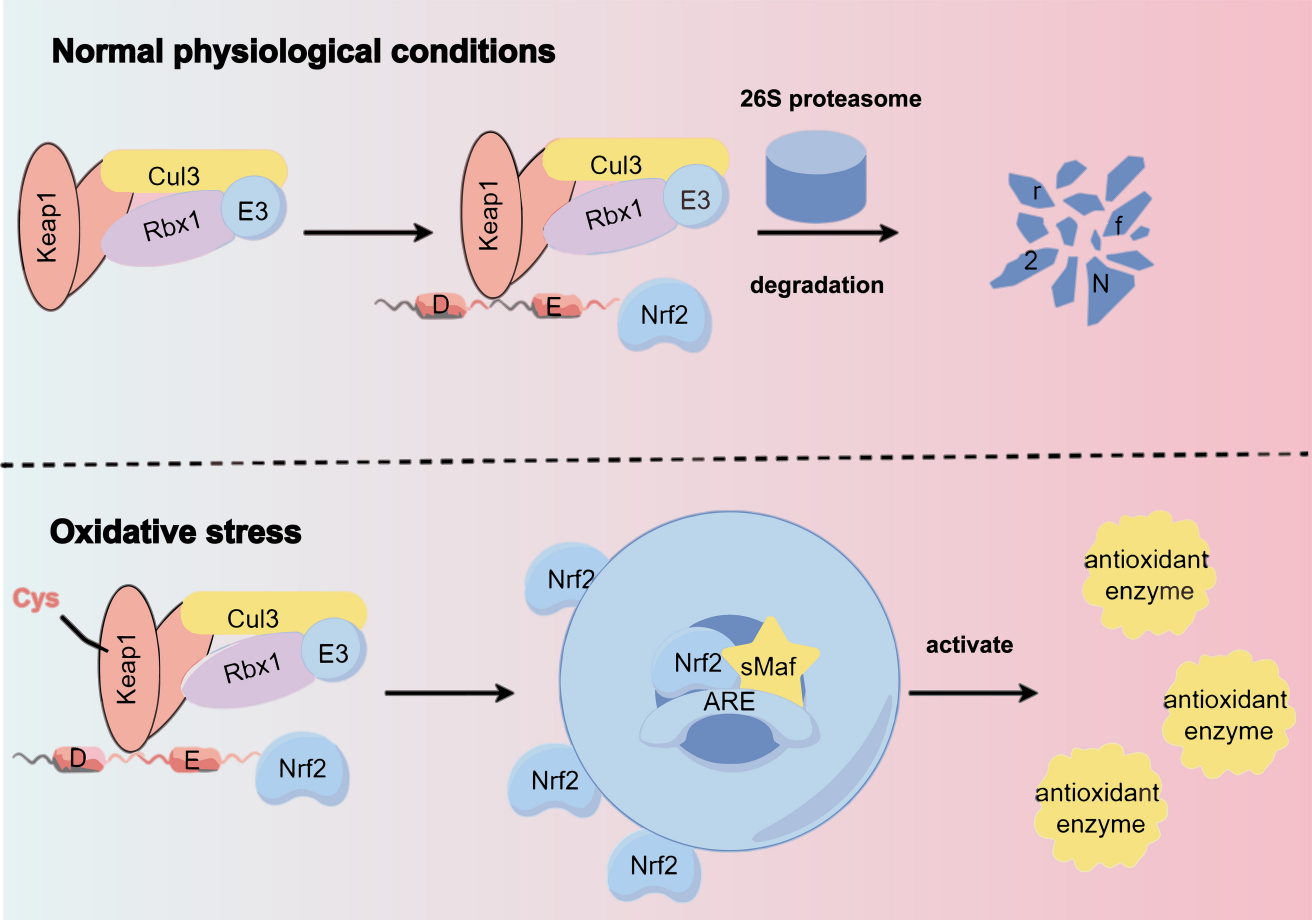
Under normal physiological conditions, the Keap1 complex binds to Nrf2, promoting its ubiquitination and subsequent degradation by the 26S proteasome. However, oxidative stress alters specific cysteine residues in Keap1, causing a conformational change that disrupts Nrf2 ubiquitination. This allows Nrf2 to translocate to the nucleus and bind to the antioxidant response element (ARE) of target genes via heterodimerization with sMAF protein, inducing the expression of cellular protective genes.

SFN (sulforaphane), is an isothiocyanate produced through glucosidase hydrolysis in cruciferous plants, known for its antioxidant properties (58). Nrf2 is a key transcription factor in the oxidative stress response, binding to the antioxidant response element (ARE) in the promoter regions of cytoprotective genes. Normally, nuclear factor erythroid 2-related factor 2 (Nrf2) is regulated by the Keap1 complex and degraded in the cytoplasm. However, under oxidative stress, Nrf2 dissociates from Keap1, avoiding degradation. This stabilized Nrf2 then enters the nucleus, forms heterodimers with Maf protein, and activates genes associated with antioxidant and detoxification by binding to AREs. This action reduces ROS levels and alleviates renal damage caused by oxidative stress (59) (**Figure 3**).

Liu et al. found that SFN significantly lowers Toll-like receptor 4 (TLR4) and interferon regulatory factor 1 (IRF1) levels in a CaOx kidney stone model by activating Nrf2. This activation promotes M2 macrophage activation, which can help reduce renal inflammatory injury and aid in preventing kidney stones (60). Additionally, SFN has been noted to encourage mitochondrial biogenesis through the Nrf2-PGC-1α (peroxisome proliferator-activated receptor gamma cofactor 1α) signaling pathway. This enhances mitochondrial number and function in reaction to damage caused by OS (61).

## 6 Conclusion

The link between ROS and the formation of CaOx has led to deep reflection on the pathogenesis of urinary stones. First, the presence of ROS may be a primary driving factor of oxidative stress in the urinary system. Their highly reactive nature allows interactions with *in vivo* biomolecules, leading to oxidative damage and inflammatory responses (62). These effects can intensify the crystallization of calcium oxalate in urine, thus creating conducive conditions for stone formation (63). Secondly, the relationship between ROS and the formation of urinary stones highlights the key role of redox balance in maintaining urinary stability and preventing stone formation. Oxidative stress disturbs this balance, creating an unstable cellular environment that can damage DNA, proteins, and lipids, as well as cause inflammation and harm to renal epithelial cells (64). Increased oxidative stress also results in heightened urinary solute supersaturation, a key factor in kidney stone formation (65).

Further research indicates that dietary and lifestyle factors significantly influence the generation and elimination of ROS. Habits such as smoking, excessive alcohol consumption, and having a sedentary lifestyle can lead to OS, which is an imbalance between the production of ROS and the body’s antioxidant defenses (66). Diets high in salt and fat, low in fiber, along with insufficient physical activity and other poor lifestyle habits, may exacerbate this stress, thus heightening the risk of calcium oxalate stones (67, 68). For those predisposed to stone formation, dietary and lifestyle modifications can be crucial in reducing ROS levels and the associated risk (69).

Additionally, the ROS-urinary stone connection offers insights for new therapeutic approaches. Exploring the potential of ROS scavengers or antioxidants in preventing and treating CaOx holds promise for innovative clinical treatment directions. This therapeutic strategy, by alleviating oxidative stress and blocking key stages in stone formation, could offer more effective and personalized treatment options for patients.

Antioxidants, compounds that aid in the elimination of ROS, have wide-ranging prospects for kidney health maintenance. Firstly, various antioxidants like vitamin E, flavonoids, and polyphenols have demonstrated efficacy in counteracting oxidative stress and reducing its impact on the body (70). By scavenging free radicals and other reactive oxygen molecules, antioxidants help to minimize oxidative harm to nucleic acids, proteins, and cell membranes, thus preserving the structural and functional integrity of kidney cells. Secondly, antioxidants may play a positive role in inhibiting inflammatory responses (71). Inflammation is an essential element in the progression of renal diseases, and antioxidants could potentially alleviate the extent of renal tissue damage by dampening the inflammation induced by oxidative stress (72). Furthermore, antioxidants may positively influence renal vascular health (73). Since oxidative stress is associated with vascular dysfunction, the application of antioxidants could protect the renal microcirculation by maintaining endothelial function and reducing vascular permeability, thereby helping to prevent some vascular complications related to kidney diseases.

Antioxidants hold significant potential in promoting kidney health. By improving the effectiveness of the body’s antioxidant defense system, antioxidants are expected to mitigate cell damage and inhibit inflammatory responses, thereby slowing the formation of CaOx. Future research should aim to unravel the specific mechanisms of various antioxidants in the kidney, focusing on their influence on cell signaling pathways, gene expression, and the modulation of inflammatory mediators. This will help clarify the precise impact of antioxidants on kidney health. Additionally, detailed investigations into how ROS contribute to the formation of calcium oxalate stones are essential. These studies will offer a more comprehensive view of the impact of oxidative stress on the urinary tract system and aid in the development of precise and targeted interventions.

## References

[1] Howles SA Thakker RV. Genetics of kidney stone disease. Nat Rev Urol. (2020) 17:407–21. doi: 10.1038/s41585-020-0332-x 32533118

[2] Stamatelou K Goldfarb DS. Epidemiology of kidney stones. Healthcare. (2023) 11:424. doi: 10.3390/healthcare11030424 36766999 PMC9914194

[3] Wigner P Grębowski R Bijak M Szemraj J Saluk-Bijak J. The molecular aspect of nephrolithiasis development. Cells. (2021) 10:1926. doi: 10.3390/cells10081926 34440695 PMC8393760

[4] Khan SR Pearle MS Robertson WG Gambaro G Canales BK Doizi S. Kidney stones. Nat Rev Dis Primer. (2016) 2:16008. doi: 10.1038/nrdp.2016.8 PMC5685519 27188687

[5] Güzel R Yildirim Ü Sarica K. Contemporary minimal invasive surgical management of stones in children. Asian J Urol. (2023) 10:239–45. doi: 10.1016/j.ajur.2023.02.001 PMC10394283 37538162

[6] Liu H Ye T Yang X Liu J Jiang K Lu H. H19 promote calcium oxalate nephrocalcinosis-induced renal tubular epithelial cell injury via a ceRNA pathway. EBioMedicine. (2019) 50:366–78. doi: 10.1016/j.ebiom.2019.10.059 PMC6921206 31735555

[7] Su L Zhang J Gomez H Kellum JA Peng Z. Mitochondria ROS and mitophagy in acute kidney injury. Autophagy. (2023) 19:401–14. doi: 10.1080/15548627.2022.2084862 PMC9851232 35678504

[8] Mulay SR Eberhard JN Desai J Marschner JA Kumar SVR Weidenbusch M. Hyperoxaluria requires TNF receptors to initiate crystal adhesion and kidney stone disease. J Am Soc Nephrol. (2017) 28:761–8. doi: 10.1681/ASN.2016040486 PMC5328164 27612997

[9] Fraternale A Zara C De Angelis M Nencioni L Palamara AT Retini M. Intracellular redox-modulated pathways as targets for effective approaches in the treatment of viral infection. Int J Mol Sci. (2021) 22:3603. doi: 10.3390/ijms22073603 33808471 PMC8036776

[10] Dröge W. Free radicals in the physiological control of cell function. Physiol Rev. (2002) 82:47–95. doi: 10.1152/physrev.00018.2001 11773609

[11] Hernansanz-Agustín P Enríquez JA. Generation of reactive oxygen species by mitochondria. Antioxidants. (2021) 10:415. doi: 10.3390/antiox10030415 33803273 PMC8001687

[12] Rudolf J Raad H Taieb A Rezvani HR. NADPH oxidases and their roles in skin homeostasis and carcinogenesis. Antioxid Redox Signal. (2018) 28:1238–61. doi: 10.1089/ars.2017.7282 28990413

[13] Li A Wang J Zhu D Zhang X Pan R Wang R. Arctigenin suppresses transforming growth factor-β1-induced expression of monocyte chemoattractant protein-1 and the subsequent epithelial–mesenchymal transition through reactive oxygen species-dependent ERK/NF-κB signaling pathway in renal tubular epithelial cells. Free Radic Res. (2015) 49:1095–113. doi: 10.3109/10715762.2015.1038258 25968940

[14] Rashed T Menon M Thamilselvan S. Molecular mechanism of oxalate-induced free radical production and glutathione redox imbalance in renal epithelial cells: effect of antioxidants. Am J Nephrol. (2004) 24:557–68. doi: 10.1159/000082043 15539792

[15] Zhang Y Murugesan P Huang K Cai H. NADPH oxidases and oxidase crosstalk in cardiovascular diseases: novel therapeutic targets. Nat Rev Cardiol. (2020) 17:170–94. doi: 10.1038/s41569-019-0260-8 PMC7880919 31591535

[16] Ray PD Huang B-W Tsuji Y. Reactive oxygen species (ROS) homeostasis and redox regulation in cellular signaling. Cell Signal. (2012) 24:981–90. doi: 10.1016/j.cellsig.2012.01.008 PMC3454471 22286106

[17] Mittler R. ROS are good. Trends Plant Sci. (2017) 22:11–9. doi: 10.1016/j.tplants.2016.08.002 27666517

[18] Di Meo S Reed TT Venditti P Victor VM. Role of ROS and RNS sources in physiological and pathological conditions. Oxid Med Cell Longev. (2016) 2016:1245049. doi: 10.1155/2016/1245049 27478531 PMC4960346

[19] Bisbal C Lambert K Avignon A. Antioxidants and glucose metabolism disorders. Curr Opin Clin Nutr Metab Care. (2010) 13:439–46. doi: 10.1097/MCO.0b013e32833a5559 20495454

[20] Abhishek A Benita S Kumari M Ganesan D Paul E Sasikumar P. Molecular analysis of oxalate-induced endoplasmic reticulum stress mediated apoptosis in the pathogenesis of kidney stone disease. J Physiol Biochem. (2017) 73:561–73. doi: 10.1007/s13105-017-0587-8 28875258

[21] Sies H. Oxidative stress: a concept in redox biology and medicine. Redox Biol. (2015) 4:180–3. doi: 10.1016/j.redox.2015.01.002 PMC4309861 25588755

[22] Tsujihata M. Mechanism of calcium oxalate renal stone formation and renal tubular cell injury. Int J Urol. (2008) 15:115–20. doi: 10.1111/j.1442-2042.2007.01953.x 18269444

[23] Joshi S Wang W Peck AB Khan SR. Activation of the NLRP3 inflammasome in association with calcium oxalate crystal induced reactive oxygen species in kidneys. J Urol. (2015) 193:1684–91. doi: 10.1016/j.juro.2014.11.093 PMC4406847 25437532

[24] Liu Y Sun Y Kang J He Z Liu Q Wu J. Role of ROS-induced NLRP3 inflammasome activation in the formation of calcium oxalate nephrolithiasis. Front Immunol. (2022) 13:818625. doi: 10.3389/fimmu.2022.818625 35154136 PMC8828488

[25] Kumar P Laurence E Crossman DK Assimos DG Murphy MP Mitchell T. Oxalate disrupts monocyte and macrophage cellular function via Interleukin-10 and mitochondrial reactive oxygen species (ROS) signaling. Redox Biol. (2023) 67:102919. doi: 10.1016/j.redox.2023.102919 37806112 PMC10565874

[26] Huang H-S Ma M-C. High sodium-induced oxidative stress and poor anticrystallization defense aggravate calcium oxalate crystal formation in rat hyperoxaluric kidneys. PloS One. (2015) 10:e0134764. doi: 10.1371/journal.pone.0134764 26241473 PMC4524621

[27] Khan SR Joshi S Wang W Peck AB. Regulation of macromolecular modulators of urinary stone formation by reactive oxygen species: Transcriptional study in an animal model of hyperoxaluria. Am J Physiol-Ren Physiol. (2014) 306:F1285–95. doi: 10.1152/ajprenal.00057.2014 PMC4042108 24598804

[28] Chaiyarit S Thongboonkerd V. Oxidative modifications switch modulatory activities of urinary proteins from inhibiting to promoting calcium oxalate crystallization, growth, and aggregation. Mol Cell Proteomics. (2021) 20:100151. doi: 10.1016/j.mcpro.2021.100151 34562649 PMC8551538

[29] Yu L Gan X Liu X An R. Calcium oxalate crystals induces tight junction disruption in distal renal tubular epithelial cells by activating ROS/Akt/p38 MAPK signaling pathway. Ren Fail. (2017) 39:440–51. doi: 10.1080/0886022X.2017.1305968 PMC6014313 28335665

[30] Hęś M Dziedzic K Górecka D Jędrusek-Golińska A Gujska E. Aloe vera (L.) webb.: natural sources of antioxidants – A review. Plant Foods Hum Nutr. (2019) 74:255–65. doi: 10.1007/s11130-019-00747-5 PMC6684795 31209704

[31] Sies H. Oxidative stress: oxidants and antioxidants. Exp Physiol. (1997) 82:291–5. doi: 10.1113/expphysiol.1997.sp004024 9129943

[32] Neha K Haider MR Pathak A Yar MS. Medicinal prospects of antioxidants: A review. Eur J Med Chem. (2019) 178:687–704. doi: 10.1016/j.ejmech.2019.06.010 31228811

[33] Liang J Gao Y Feng Z Zhang B Na Z Li D. Reactive oxygen species and ovarian diseases: Antioxidant strategies. Redox Biol. (2023) 62:102659. doi: 10.1016/j.redox.2023.102659 36917900 PMC10023995

[34] Traber MG Atkinson J. antioxidant and nothing more. Free Radic Biol Med. (2007) 43:4–15. doi: 10.1016/j.freeradbiomed.2007.03.024 17561088 PMC2040110

[35] Miller RA Britigan BE. Role of oxidants in microbial pathophysiology. Clin Microbiol Rev. (1997) 10(1):1–18 doi: 10.1128/CMR.10.1.1 PMC172912 8993856

[36] Vertuani S Angusti A Manfredini S. The antioxidants and pro-antioxidants network: an overview. Curr Pharm Des. (2004) 10:1677–94. doi: 10.2174/1381612043384655 15134565

[37] Zhang P Li T Wu X Nice EC Huang C Zhang Y. Oxidative stress and diabetes: antioxidative strategies. Front Med. (2020) 14:583–600. doi: 10.1007/s11684-019-0729-1 32248333

[38] Allan K Kelly FJ Devereux G. Antioxidants and allergic disease: a case of too little or too much? Clin Exp Allergy. (2010) 40:370–80. doi: 10.1111/j.1365-2222.2009.03413.x 19968654

[39] Birben E Sahiner UM Sackesen C Erzurum S Kalayci O. Oxidative stress and antioxidant defense. World Allergy Organ J. (2012) 5:9–19. doi: 10.1097/WOX.0b013e3182439613 23268465 PMC3488923

[40] Bouayed J Bohn T. Exogenous Antioxidants—Double-Edged Swords in Cellular Redox State: Health Beneficial Effects at Physiologic Doses versus Deleterious Effects at High Doses. Oxid Med Cell Longev. (2010) 3:228–37. doi: 10.4161/oxim.3.4.12858 PMC2952083 20972369

[41] Liu C-C Wu C-F Lee Y-C Huang T-Y Huang S-T Wang H-S. Genetic polymorphisms of mnSOD modify the impacts of environmental melamine on oxidative stress and early kidney injury in calcium urolithiasis patients. Antioxidants. (2022) 11:152. doi: 10.3390/antiox11010152 35052656 PMC8773063

[42] Zelko IN Mariani TJ Folz RJ. Superoxide dismutase multigene family: a comparison of the CuZn-SOD (SOD1), Mn-SOD (SOD2), and EC-SOD (SOD3) gene structures, evolution, and expression. Free Radic Biol Med. (2002) 33:337–49. doi: 10.1016/S0891-5849(02)00905-X 12126755

[43] Nozik-Grayck E Suliman HB Piantadosi CA. Extracellular superoxide dismutase. Int J Biochem Cell Biol. (2005) 37:2466–71. doi: 10.1016/j.biocel.2005.06.012 16087389

[44] Rubino V La Rosa G Pipicelli L Carriero F Damiano S Santillo M. Insights on the multifaceted roles of wild-type and mutated superoxide dismutase 1 in amyotrophic lateral sclerosis pathogenesis. Antioxidants. (2023) 12:1747. doi: 10.3390/antiox12091747 37760050 PMC10525763

[45] Fukai T Ushio-Fukai M. Superoxide dismutases: role in redox signaling, vascular function, and diseases. Antioxid Redox Signal. (2011) 15:1583–606. doi: 10.1089/ars.2011.3999 PMC3151424 21473702

[46] Kang J Sun Y Deng Y Liu Q Li D Liu Y. Autophagy-endoplasmic reticulum stress inhibition mechanism of superoxide dismutase in the formation of calcium oxalate kidney stones. BioMed Pharmacother. (2020) 121:109649. doi: 10.1016/j.biopha.2019.109649 31733571

[47] Chaiyarit S Thongboonkerd V. Mitochondrial dysfunction and kidney stone disease. Front Physiol. (2020) 11:566506. doi: 10.3389/fphys.2020.566506 33192563 PMC7606861

[48] Albert A Paul E Rajakumar S Saso L. Oxidative stress and endoplasmic stress in calcium oxalate stone disease: the chicken or the egg? Free Radic Res. (2020) 54:244–53. doi: 10.1080/10715762.2020.1751835 32292073

[49] Blaner WS Shmarakov IO Traber MG. Vitamin A and vitamin E: will the real antioxidant please stand up? Annu Rev Nutr. (2021) 41:105–31. doi: 10.1146/annurev-nutr-082018-124228 34115520

[50] Thamilselvan S Menon M. Vitamin E therapy prevents hyperoxaluria-induced calcium oxalate crystal deposition in the kidney by improving renal tissue antioxidant status. BJU Int. (2005) 96:117–26. doi: 10.1111/j.1464-410X.2005.05579.x 15963133

[51] Chmiel JA Stuivenberg GA Al KF Akouris PP Razvi H Burton JP. Vitamins as regulators of calcium-containing kidney stones — new perspectives on the role of the gut microbiome. Nat Rev Urol. (2023) 20:615–37. doi: 10.1038/s41585-023-00768-5 PMC10169205 37161031

[52] Fryer MJ. Vitamin E as a protective antioxidant in progressive renal failure. Nephrology. (2000) 5:1–7. doi: 10.1046/j.1440-1797.2000.00504.x

[53] Gramza A Korczak J Amarowicz R. Tea polyphenols – their antioxidant properties and biological activity – a review.

[54] Wiseman SA Balentine DA Frei B. Antioxidants in tea. Crit Rev Food Sci Nutr. (1997) 37:705–18. doi: 10.1080/10408399709527798 9447271

[55] Yan Z Zhong Y Duan Y Chen Q Li F. Antioxidant mechanism of tea polyphenols and its impact on health benefits. Anim Nutr. (2020) 6:115–23. doi: 10.1016/j.aninu.2020.01.001 PMC7283370 32542190

[56] Kumar Sen A Sen DB Zanwar AS Balaraman R Shah U Maheshwari RA. atechins and theaflavins: an overview on therapeutic application. J Nat Remedies. (2022), 330–46. doi: 10.18311/jnr/2022/30181

[57] Ye T Yang X Liu H Lv P Lu H Jiang K. Theaflavin protects against oxalate calcium-induced kidney oxidative stress injury via upregulation of SIRT1. Int J Biol Sci. (2021) 17:1050–60. doi: 10.7150/ijbs.57160 PMC8040307 33867828

[58] Chang R. Research advances in the protective effect of sulforaphane against kidney injury and related mechanisms. Bio Web Conf. (2022) 55:1006. doi: 10.1051/bioconf/20225501006

[59] Wei W Ma N Fan X Yu Q Ci X. The role of Nrf2 in acute kidney injury: Novel molecular mechanisms and therapeutic approaches. Free Radic Biol Med. (2020) 158:1–12. doi: 10.1016/j.freeradbiomed.2020.06.025 32663513

[60] Liu H Yang X Tang K Ye T Duan C Lv P. Sulforaphane elicts dual therapeutic effects on Renal Inflammatory Injury and crystal deposition in Calcium Oxalate Nephrocalcinosis. Theranostics. (2020) 10:7319–34. doi: 10.7150/thno.44054 PMC7330860 32641994

[61] Huang J Liang Y Zhou L. Natural products for kidney disease treatment: Focus on targeting mitochondrial dysfunction. Front Pharmacol. (2023) 14:1142001. doi: 10.3389/fphar.2023.1142001 37007023 PMC10050361

[62] Mittal M Siddiqui MR Tran K Reddy SP Malik AB. Reactive oxygen species in inflammation and tissue injury. Antioxid Redox Signal. (2014) 20:1126–67. doi: 10.1089/ars.2012.5149 PMC3929010 23991888

[63] Khan SR. Reactive oxygen species, inflammation and calcium oxalate nephrolithiasis. Transl Androl Urol. (2014), 3. 10.3978/j.issn.2223-4683.2014.06.04 PMC4220551 25383321

[64] Farhood B Najafi M Salehi E Hashemi Goradel N Nashtaei MS Khanlarkhani N. Disruption of the redox balance with either oxidative or anti-oxidative overloading as a promising target for cancer therapy. J Cell Biochem. (2019) 120:71–6. doi: 10.1002/jcb.27594 30203529

[65] Bird VY Khan SR. How do stones form? Is unification of theories on stone formation possible?. Arch Esp Urol (2018) 70(1):12–27. PMC5683182 28221139

[66] Sharifi-Rad M Anil Kumar NV Zucca P Varoni EM Dini L Panzarini E. Lifestyle, oxidative stress, and antioxidants: back and forth in the pathophysiology of chronic diseases. Front Physiol. (2020) 11:694. doi: 10.3389/fphys.2020.00694 32714204 PMC7347016

[67] Nouvenne A Ticinesi A Morelli I Guida L Borghi L Meschi T. Fad diets and their effect on urinary stone formation. Transl Androl Urol. (2014) 3(3):303-12. doi: 10.3978/j.issn.2223-4683.2014.06.01 PMC4708571 26816783

[68] Nawaz N Tahir H Tamiz H Basharat S Hassan M Aamir M. Association between dietary practices and calcium oxalate stone formation in urinary tract among the patients of urolithiasis. (2020) 3:.

[69] Khan SR. Is oxidative stress, a link between nephrolithiasis and obesity, hypertension, diabetes, chronic kidney disease, metabolic syndrome? Urol Res. (2012) 40:95–112. doi: 10.1007/s00240-011-0448-9 22213019 PMC5683185

[70] Pizzino G Irrera N Cucinotta M Pallio G Mannino F Arcoraci V. Oxidative stress: harms and benefits for human health. Oxid Med Cell Longev. (2017) 2017:8416763. doi: 10.1155/2017/8416763 28819546 PMC5551541

[71] Arulselvan P Fard MT Tan WS Gothai S Fakurazi S Norhaizan ME. Role of antioxidants and natural products in inflammation. Oxid Med Cell Longev. (2016) 2016:5276130. doi: 10.1155/2016/5276130 27803762 PMC5075620

[72] Hong YA Park CW. Catalytic antioxidants in the kidney. Antioxidants. (2021) 10:130. doi: 10.3390/antiox10010130 33477607 PMC7831323

[73] Dennis J Witting P. Protective role for antioxidants in acute kidney disease. Nutrients. (2017) 9:718. doi: 10.3390/nu9070718 28686196 PMC5537833

